# HPV infection and 5mC/5hmC epigenetic markers in penile squamous cell carcinoma: new insights into prognostics

**DOI:** 10.1186/s13148-022-01360-1

**Published:** 2022-10-25

**Authors:** Renan da Silva Santos, Carlos Gustavo Hirth, Daniel Pascoalino Pinheiro, Maria Júlia Barbosa Bezerra, Isabelle Joyce de Lima Silva-Fernandes, Dayrine Silveira de Paula, Ana Paula Negreiros Nunes Alves, Manoel Odorico de Moraes Filho, Arlindo de Alencar Araripe Moura, Marcos Venício Alves Lima, Claudia do Ó Pessoa, Cristiana Libardi Miranda Furtado

**Affiliations:** 1 Drug Research and Development Center, Department of Physiology and Pharmacology, Federal University of Ceará, Fortaleza, Brazil; 2 Laboratory of Pathology, Cancer Institute of Ceará, Fortaleza, Brazil; 3 Laboratory of Molecular Biology and Genetics, Cancer Institute of Ceará, Fortaleza, Brazil; 4 Department of Animal Science, Federal University of Ceará, Fortaleza, Brazil; 5 Drug Research and Development Center, Postgraduate Program in Translational Medicine, Federal University of Ceará, Fortaleza, Brazil; 6 Experimental Biology Center, University of Fortaleza, Fortaleza, Brazil; 7 Department of Dental Clinic, Faculty of Pharmacy, Dentistry and Nursing, Federal University of Ceará, Fortaleza, Brazil

**Keywords:** Penile cancer, Human papillomavirus, p16^INK4a^, High-risk HPV, Global DNA methylation, Global DNA hydroxymethylation

## Abstract

**Background:**

Penile cancer is one of the most aggressive male tumors. Although it is preventable, the main etiologic causes are lifestyle behaviors and viral infection, such as human papillomavirus (HPV). Long-term epigenetic changes due to environmental factors change cell fate and promote carcinogenesis, being an important marker of prognosis. We evaluated epidemiological aspects of penile squamous cell carcinoma (SCC) and the prevalence of HPV infection using high-risk HPV (hrHPV) and p16^INK4A^ expression of 224 participants. Global DNA methylation was evaluated through 5-methylcytosine (5mC) and 5-hydroxymethylcytosine (5hmC).

**Results:**

The incidence of HPV was 53.2% for hrHPV and 22.32% for p16^INK4a^. hrHPV was not related to systemic or lymph node metastasis and locoregional recurrence, nor influenced the survival rate. P16^INK4a^ seems to be a protective factor for death, which does not affect metastasis or tumor recurrence. Lymph node and systemic metastases and locoregional recurrence increase the risk of death. An increased 5mC mark was observed in penile SCC regardless of HPV infection. However, there is a reduction of the 5hmC mark for p16^INK4a^ + (*P* = 0.024). Increased 5mC/5hmC ratio (> 1) was observed in 94.2% of penile SCC, irrespective of HPV infection. Despite the increase in 5mC, it seems not to affect the survival rate (HR = 1.06; 95% CI 0.33–3.38).

**Conclusions:**

P16^INK4a^ seems to be a good prognosis marker for penile SCC and the increase in 5mC, an epigenetic mark of genomic stability, may support tumor progression leading to poor prognosis.

**Supplementary Information:**

The online version contains supplementary material available at 10.1186/s13148-022-01360-1.

## Background

Penile cancer is a rare tumor type with an increased worldwide incidence, having 36,068 new cases registered in 2020. The majority of cases occur in regions with a low human development index, where India (10,677), China (4,628) and Brazil (1,698) are the most affected countries [1]. Despite the low incidence compared to other types of male malignancies, penile cancer has a poor prognosis and is associated with high mortality [2] and morbidity [3]. Therapeutic strategies are very limited and, therefore, the main related therapy is partial or total penectomy, which directly affects the emotional and social life of patients. Given narrow options for early diagnosis and non-surgery treatments, restricted funding for medical care, and mutilating treatments resulting in negative effects on well-being, penile cancer can be regarded as a neglected disease [4–6].

Different histological types are associated with penile cancer, such as sarcoma, melanoma and basal cell carcinoma [7, 8], nevertheless, penile squamous cell carcinoma (SCC) is reported in 95% of cases worldwide [9, 10]. Multiple risk factors are described, mostly related to lifestyle behaviors, such as promiscuous sexual behavior [11], history of zoophilia [12], poor hygiene [13], psoralen UV-A phototherapy [14], smoking [15] and obesity [16]. Non-circumcision (phimosis) leads to chronic inflammation conditions like posthitis, lichen sclerosus and balanitis xerotic obliterans [9] increasing the risk of developing penile cancer by 22-fold [17]. However, human papillomavirus (HPV) infection in penile cancer is one of the main etiologic causes, especially for squamous cell carcinomas [18].

The prevalence of HPV in penile neoplasia can vary widely depending on the literature and among different regions of the world, ranging from 11 to 87% [19, 20]. Associations of HPV infection and death risk are still unclear, as the results are controversial [21–23]. Positive survival prognosis for high-risk HPV (hrHPV) in penile cancer has already been highlighted [24] and Wang and collaborators (2020) [25] have recently suggested a regional lymph node infiltration staging based on the presence of hrHPV. Subtypes of hrHPV are linked to malignant lesions due to the degradation of the cell cycle control proteins P53 and Rb, and the expression of the viral oncoproteins, HPV E6 and HPV E7, which causes evasion to cell death and DNA damage repair, respectively [26]. These viral oncoproteins' expression promotes chromosomal rearrangements, multiple centromeres and aneuploidy [27]. Viral infection, as HPV is often related to an increase in genomic instability by aberrantly reprogramming the epigenome [28]. In different squamous cell carcinomas hrHPV directly modulates enzymes that maintain the conformation of nucleosomes [28, 29] and enzymes responsible for maintaining DNA methylation [30, 31].

Environmentally induced epigenetic changes have been recently added as hallmarks of cancer, which contribute to tumor initiation and progression [32]. Global DNA methylation is characterized by the addition of a methyl at position 5 of cytosine in a dinucleotide CpG (cytosine-phosphate-guanine) resulting in a 5-methylcytosine (5mC) [33], which affects gene expression, chromatin remodeling and genomic stability. DNA methyltransferases (DNMTs) mediate the transfer of the methyl group to DNA, and loss of the 5mC marker can occur either passively by DNA replication or actively by the enzymes known as Ten-Eleven-Translocation (TETs) [34]. These enzymes catalyze the conversion of 5mC into 5-hydroxymethylcytosine (5hmC). Imbalances between 5mC and 5hmC marks cause transcriptional dysregulation of promoters and enhancers, changing the cell fate [35]. The imbalance of 5mc/5hmc dynamics has been described in many types of cancers, but not for penile squamous cell carcinomas.

Since HPV infection may drive epigenetic changes, the characterization of the global DNA methylation and its association with hrHPV DNA and p16^INK4a^ expression will provide better knowledge about the molecular mechanisms related to penile SCC. Therefore, evaluate the prevalence of HPV infection and 5mC and 5hmC epigenetic marks in penile SCC and its association with clinicopathological alterations. As an important mechanism of genomic stability, aberrant epigenetic reprogramming related to penile SCC pathogenesis and viral infection may be used as a biomarker for prognosis and targeted therapies. Determining the prevalence of HPV, one of the most common infections of the reproductive tract, is important for the development of public health strategies for the prevention and treatment of related diseases.

## Results

### Clinicopathological characteristics and HPV infection

The participants’ age, HPV infection, pathological classification, and treatments of penile SCC are presented in Table 1. Participants' mean age was 63.8 (± 15.86) years old, and the incidence of SCC was higher in men over 60 years old (59.2%; 133/224), even though the manifestation of the disease was also detected before the age of 60 (33.2%; 74/224) and 40 (7.6%; 17/224). The frequency of HPV infection was 22.3% when evaluated with p16^INK4a^ immunodepression and reached 53.2% when tested by hrHPV hybridization. Low-risk HPV infection was not identified. Tumor staging based on the primary tumor was mainly corpus spongiosum invasion (39.3%, pT2) followed by the first stages including carcinoma in situ (pTis), noninvasive carcinoma (pTa), subepithelial invasion without lymphovascular invasion (pT1a) and with lymphovascular invasion (pT1b) (30.1%).

**Table 1:** Clinical and pathological aspects, staging and treatment of penile squamous cell carcinoma participants

	N (%)
Age (years)	
Mean (range)	63.8 years (18–103)
< 40	17/224 (7.6)
40–60	74/224 (33.2)
> 60	133/224 (59.2)
p16^INK4a^	
No	174/224 (77.7)
Yes	50/224 (22.3)
hrHPV	
No	88/188 (46.8)
Yes	100/188 (53.2)
Staging	
pTis + pTa + pT1(a.b)	66/219 (30.1)
pT2	86/219 (39.3)
pT3	62/219 (28.3)
pT4	5/219 (2.3)
Sistemic metastasis	
No	157/188 (83.5)
Yes	31/188 (16.5)
Lymph node metastasis	
No	124/207 (59.9)
Yes	83/207 (40.1)
Locoregional recurrence	
No	139/188 (73.9)
Yes	49/188 (26.1)
PLI	
Mild and Moderate	130/217 (59.9)
Intense	87/217 (40.1)
PPI	
Mild and Moderate	205/217 (94.5)
Intense	12/217 (5.5)
ILI	
Mild and Moderate	215/217 (99.1)
Intense	2/217 (0.9)
IPI	
Mild and Moderate	127/217 (58.5)
Intense	90/217 (41.5)
Surgical procedure	
Partial amputation	164/224 (73.2)
Total amputation	33/224 (14.7)
Others	27/224 (12.1)
Chemotherapy	
No	144/194 (74.2)
Yes	50/194 (25.8)
Radiotherapy	
No	165/192 (85.9)
Yes	27/192 (14.1)

*pTis* carcinoma in situ, *pTa* noninvasive carcinoma, *pT1a* subepithelial invasion without lymphovascular invasion, perineural invasion or grade 3, *pT1b* subepithelial invasion with lymphovascular invasion, perineural invasion or grade 3, *pT2* invasion of corpus spongiosum, *pT3* invasion of corpus cavernosum, *pT4* invasion of adjacent structures including scrotum, prostate and pubic bone, *PLI* peritumoral lymphocyte infiltrate, *PPI* peritumoral polymorphonuclear infiltrate, *ILI* intratumoral lymphocyte infiltrate, *IPI* intratumoral polymorphonuclear infiltrate

Systemic metastasis was observed in 16.5% of participants diagnosed with penile SCC, while lymph node metastasis was presented in 40.1% and locoregional recurrence in 26.1%. Intense peritumoral lymphocyte infiltrate (PLI) and intratumoral polymorphonuclear infiltrate (IPI) reached 40.1% and 41.5% of penile SCC, respectively. The main therapeutic strategies were partial amputation 73.2% and total amputation 14.7%, followed by other associated strategies (12.1%) such as prostatectomy, emasculation, and exercise injury. Chemotherapy was the treatment option for 25.8% of men, which consisted of cisplatin associated with 5-Fluorouracil or cisplatin associated with taxol or paclitaxel, as a palliative or neoadjuvant therapy before and after lymphadenectomy. Radiotherapy was used only as a palliative alternative for pain in cases of metastasis (14.1%) (Table 1). Despite the differences in HPV infection diagnosis using p16^INK4a^ or hrHPV, a positive correlation was observed between these markers (*P* = 0.003, Table 2). Radiotherapy was associated with p16^INK4a^ positive cases (*P* = 0.0136). All other clinicopathological variables were not associated with HPV markers p16^INK4a^ and hrHPV.

**Table 2:** Clinical-pathological aspects, staging and therapy of penile SCC patients and p16^INK4a^ and hrHPV correlation

	p16^INK4a*^		*P*-value	hrHPV		*P*-value
	Negative (%)	Positive (%)		Negative (%)	Positive (%)	
hrHPV						
Negative	76 (52.8)	12 (27.3)	**0.003**	–	–	
Positive	68 (47.2)	32 (72.7)				
Staging						
pTis + pTa + pT1(a.b)	50 (29.4)	16 (32.6)	0.7138	25 (29.1)	26 (26.5)	0.4003
pT2	69 (40.6)	17 (34.7)		38 (44.2)	35 (35.7)	
pT3	48 (28.3)	14 (28.6)		22 (25.6)	34 (34.7)	
pT4	3 (1.7)	2 (4.1)		1 (1.1)	3 (3.1)	
Lymph Node Metastasis						
Negative	101 (62.3)	23 (51.1)	0.1737	55 (66.3)	49 (52.1)	0.0566
Positive	61 (37.7)	22 (48.9)		28 (33.7)	45 (47.9)	
Locoregional Recurrence						
Negative	106 (72.6)	33 (78.6)	0.4374	57 (76)	60 (69.8)	0.3761
Positive	40 (27.4)	9 (21.4)		18 (24)	26 (30.2)	
Metastasis						
Negative	123 (84.2)	34 (80.9)	0.6122	62 (82.7)	72 (83.7)	0.8582
Positive	23 (15.8)	8 (19.1)		13 (17.3)	14 (16.3)	
PLI						
Mild and Moderate	99 (58.6)	31 (64.6)	0.4539	49 (58.3)	58 (59.8)	0.842
Intense	70 (41.4)	17 (35.4)		35 (41.7)	39 (40.2)	
PPI						
Mild and Moderate	157 (92.9)	48 (100)	0.0575	82 (97.6)	90 (92.8)	0.1356
Intense	12 (7.1)	0 (0)		2 (2.4)	7 (7.2)	
ILI						
Mild and Moderate	167 (98.8)	48 (100)	0.4489	83 (98.8)	96 (99)	0.9184
Intense	2 (1.2)	0 (0)		1 (1.2)	1 (1)	
IPI						
Mild and Moderate	99 (58.6)	28 (58.3)	0.9756	46 (54.8)	56 (57.7)	0.6878
Intense	70 (41.4)	20 (41.7)		38 (45.2)	41 (42.3)	
Surgical procedure						
Partial amputation	127 (73)	37 (74)	0.1441	64 (72.7)	75 (75)	0.5434
Total amputation	29 (16.7)	4 (8)		16 (18.2)	13 (13)	
Others	18 (10.3)	9 (18)		8 (9.1)	12 (12)	
Chemotherapy						
Negative	115 (76.7)	29 (65.9)	0.1514	58 (76.3)	65 (72.2)	0.5486
Positive	35 (23.3)	15 (34.1)		18 (23.7)	25 (27.8)	
Radiotherapy						
Negative	133 (89.3)	32 (74.4)	**0.0136**	65 (85.5)	76 (85.4)	0.9807
Positive	16 (10.7)	11 (25.6)		11 (14.5)	13 (14.6)	
5mC						
< 30%	2 (1.5)	0 (0)	0.528	1 (1.5)	0 (0)	0.4534
> 30–< 60%	11 (8.5)	5 (13.2)		6 (9)	10 (12)	
> 60%	116 (90)	33 (86.8)		60 (89.5)	73 (88)	
5hmC						
0%	18 (14.2)	8 (21.6)	0.1236	14 (21.2)	11 (13.5)	0.5893
< 30%	34 (26.7)	11 (29.7)		17 (25.8)	26 (31.7)	
> 30–< 60%	29 (22.8)	12 (32.5)		17 (25.8)	20 (24.4)	
> 60%	46 (36.2)	6 (16.2)		18 (27.2)	25 (30.4)	

Bold indicates *p* < 0.05

*pTis* carcinoma in situ, *pTa* noninvasive carcinoma, *pT1a* subepithelial invasion without lymphovascular invasion, perineural invasion or grade 3, *pT1b* subepithelial invasion with lymphovascular invasion, perineural invasion or grade 3, *pT2* invasion of corpus spongiosum, *pT3* invasion of corpus cavernosum, *pT4* invasion of adjacent structures including scrotum, prostate and pubic bon PLI: Peritumoral Lymphocytic Infiltrate, *PPI* peritumoral polymorphonuclear infiltrate, *ILI* intratumoral lymphocyte infiltrate, *IPI* intratumoral polymorphonuclear infiltrate, *5mC* 5-methylcytosine, *5hmC* 5-hydroxymethylcitosine

*Each column adds up to 100%

**Fig. 1:**
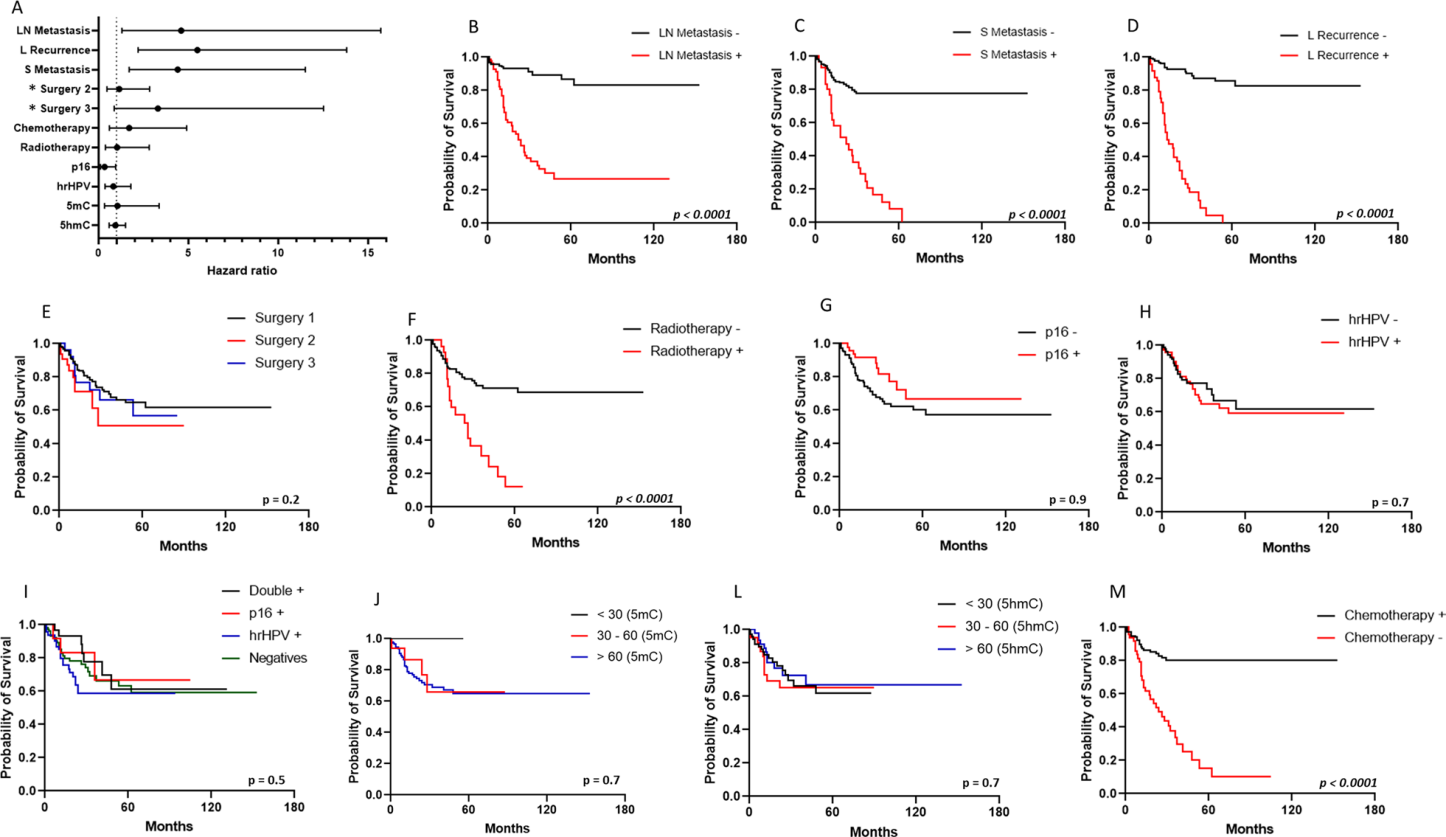
Survival analyses. Multivariate-adjusted Cox hazards regression model (**A**) and Kaplan–Meier survival curves: **B** lymph node metastasis; **C** systemic metastasis; **D** locoregional recurrence; **E** surgery; **F** radiotherapy; **G** p16 ^INK4a^; **H** hrHPV; **I** double positive (+), p16^INK4a^ (+), hrHPV (+) and negative (−); **J** 5-mehtylcitosine, 5mC; **L** 5-hydroximetilcitosine, 5hmC and **M** chemotherapy. Surgery 1, partial amputation; Surgery 2, total amputation; Surgery 3, others. *Adjusted with surgery 1

The survival rate was measured by a multivariate-adjusted Cox hazards regression model (Fig. 1A, Additional file 1: Supplementary Table 1). Locoregional recurrence (Hazard Ratio, HR = 5.51, 95% CI 2.20–13.84; *P* < 0.001), systemic metastasis (HR = 4.40, 95% CI 1.68–11.49, *P* = 0.003) and lymph node metastasis (HR = 4.65, 95% CI 1.3–15.7, *P* = 0.013) decreased the survival rate in penile SCC. On the other hand, p16^INK4a^ expression seems to be a protective factor for death (HR = 0.34, 95% CI 0.531–2.05; *P* = 0.04) and hrHPV (HR = 0.82, 95% CI 0.36–1.86; *P* = 0.637) did not affect survival even with a HR < 1. The remaining parameters evaluated did not affect the survival rate. However, on the non-adjusted Kaplan–Meier curves metastasis, recurrence, chemotherapy, radiotherapy (Fig. 1B–D, F, M) and staging (Additional file 1: Supplementary Fig. 3E) seems to decrease the time to death (*P* < 0.05).

### 5mC and 5hmC marks in penile SCC

**Fig. 2:**
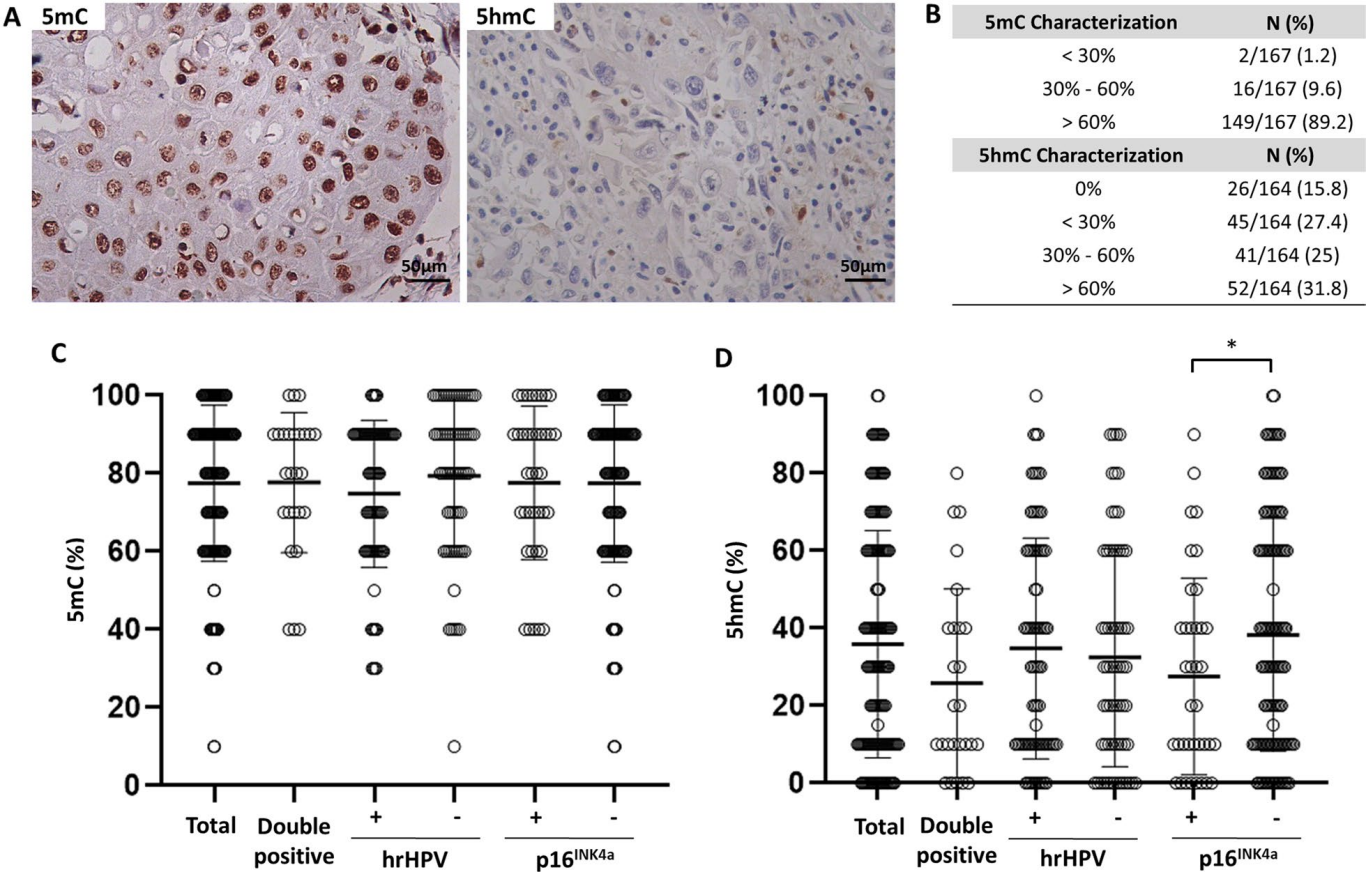
The pattern of 5mC and 5hmC epigenetic marks on penile SCC. **A** 5mC and 5hmC marks staining in penile squamous cell carcinoma. **B** Stratification of 5mC and 5hmC mark levels. Distribution of 5mC **C** and 5hmC **D** levels in a total number of samples and separated by hrHPV and p16^INK4a^ groups. Data are presented as mean and standard deviation. *P = 0.024

**Fig. 3:**
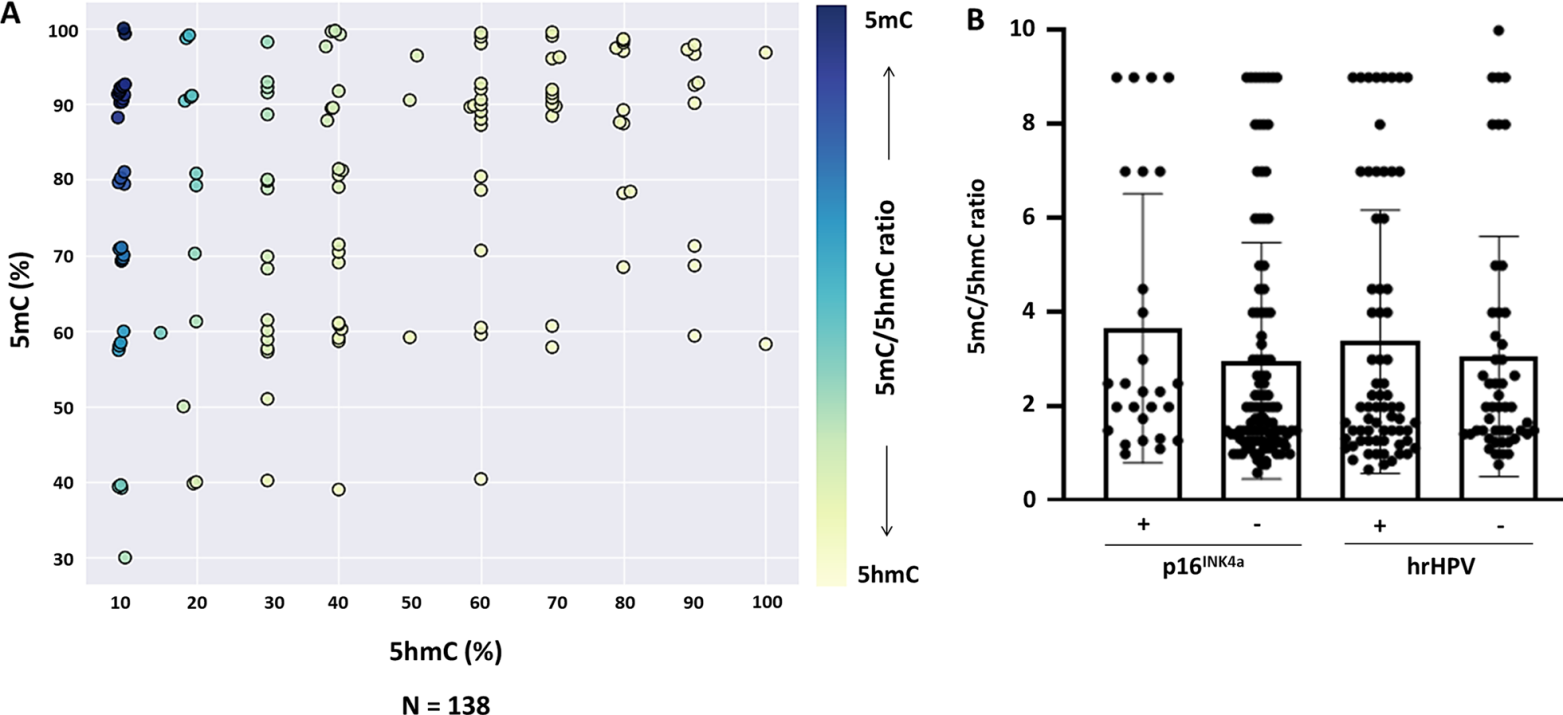
5mC/5hmC ratio distribution. **A** 5mC/5hmC ratio dispersion by 5mC and 5hmC percentage, y and x-axis, respectively. The higher the color tone, the higher the ratio value. **B** 5mC/5hmC ratio distribution values by hrHPV and p16^INK4a^ groups

Increased global 5mC (> 60%) mark was observed in 89.2% of penile SCC cases, regardless of HPV infection (Fig. 2A , B). The average of global 5hmC levels varied from lower (< 30%), intermediate (30–60%), and increased (> 60%) hydroxymethylation levels (Fig. 2B). The total cases of penile cancer evaluated for the 5mC mark (Fig. 2C), corresponding to 77.5% ± 19.9, have a higher percentage value than the 5hmC mark (35.9% ± 29.3, Fig. 2D). No association was observed between global DNA methylation marks and HPV infection (Table 2). Also, no differences were observed in the distribution of the 5mC mark for both hrHPV + and p16^INK4a^ + (Fig. 2C), but an increase in the 5hmC mark for p16^INK4a^ negative (*P* = 0.024) was observed (Fig. 2D). 5mC in double positive HPV was similar to hrHPV and p16^INK4a^ (Fig. 2C) and 5hmC double positive HPV was similar to p16^INK4a^ + (Fig. 2D). The 5mC/5hmC ratio represents the proportion between the marks for each sample (Fig. 3A). The majority of penile SCC showed 5mC/5hmC ratio greater or equal to 1 (94.2%), which was not associated with viral infection (Fig. 3B). Despite increased 5mC mark, methylation level was not associated with survival rate (HR = 1.05, 95% CI 0.33–3.38; *P* = 0.92), as well as 5hmC (HR = 0.95, 95% CI 0.60–1.50; *P* = 0.82).

## Discussion

Penile cancer is one of the most aggressive male malignancies, although it is one of the easiest to prevent, as the main etiologic causes are lifestyle conditions and viral infection [38]. We observed a high incidence of HPV infection considering hrHPV detection (53.2%) while the incidence reduces using p16^INK4a^ (22.3%). Despite the elevated incidence, HPV infection was not related to systemic or lymph node metastasis and locoregional recurrence and, therefore, does not influence the survival rate. Long-term changes in DNA methylation are characteristic of environmentally induced carcinogenesis as penile cancer [13]. Increased 5mC was observed in penile SCC, which seems to be a stable epigenetic marker, while the 5hmC was lower and more variable. The HPV infection was not related to the overall 5mC/5hmC ratio. Interestingly, 5mC may be a risk factor for poor prognosis and survival rate.

The incidence of penile cancer directly impacts public health, as the specialized medical care, psychosocial impact and the risk of cervical cancer in their female partners increase the cost of treatment [66]. Despite significant reductions in hospital admissions in Brazil over the past two decades, the incidence of penile carcinoma is still high and quite unequal across the five regions of the country, whereby the Northwest stands out with the highest incidence and the lowest per capita income in the country [39]. Socioeconomic status and low education levels reflect on penile SCC staging at diagnosis [40, 41] and, a recent study conducted in the states of Maranhão and Rio de Janeiro, Brazil, report that 69.9% of patients were diagnosed with pT2 and pT3, and 87.9% of them underwent surgical removal of the organ. A contrasting scenario is observed in developed countries, such as the USA, where 54.1% of penile SCC staging are pT1 and there were 75.8% of amputated cases [42]. Penile SCC was observed in young men, ranging from 18 to 103 years old, different from other cities in Brazil as the age varied between 23 to 98 years [13, 41], and even more discrepant in developed countries, where the disease is rare before the third decade of life [43, 44].

Despite the correlation between hrHPV and p16^INK4a^, the incidence of HPV infection was twofold higher using hrHPV than p16^INK4a^, and only 17% of all cases were double positive for both markers. Considering that the tumor suppressor protein p16^INK4a^ is upregulated by the HPV oncogene [45, 46], HPV DNA detection may be a primary event that is followed by increased expression of this protein. Also, negative testing for p16^INK4a^ expression may be linked to gene expression loss, caused by promoter methylation events and loss of heterozygosity [45, 46], implying a false negative result [49]. A recent systematic review and meta-analysis showed 49% (95% CI 43.1–54.9) of HPV prevalence using qPCR or hybridization analyses (*n* = 3772 cases in 47 studies) and 42.1% (95% CI 36.4–47.8) using p16^INK4a^ (*n* = 1296 cases in 23 studies) in SCC [47]. Both techniques are widely used for HPV detection, however, the values may show discrepancies. Therefore, double positive HPV DNA and p16^INK4a^ must be considered for some types of cancer, such as head and neck SCC [74].

Although previously mentioned as a risk factor for penile SCC [10, 23], our present analysis indicates that hrHPV and p16^INK4a^ were not related to disease staging, metastasis, or infiltration of the immune system cells. On the other hand, p16^INK4a^ seems to be a protective factor for death, as well as hrHPV (HR < 1), although it was not significant, an increase in sample size would answer this question. In addition, positive cases for p16^INK4a^ seem to be associated with radiotherapy. Although previous studies associate p16^INK4a^ expression and radiotherapy [75, 76], this might be a random correlation. Increased p16^INK4a^ was correlated with a good prognosis, as lack of nodal metastasis in head and neck SCC [67, 77] and cervical cancer [78]. In penile SCC, Martins and collaborators showed that p16^INK4a^ was not associated with prognosis parameters or survival rate [46].

The presence of lymph node metastasis in the inguinal and iliac regions increased the risk of death, which is the most common metastatic event [50] and the main prognostic marker [51]. Systemic metastases are rarely reported [41, 50] and locoregional recurrences are also less frequent [52], but these two parameters were associated with an increased risk of death by 4.3 and 5.5-fold, respectively. Penectomy is still a widely used treatment for extensive lesions or tumors involving the base and the bulbar urethral part of the penis [13, 41, 53], but our results indicate that this type of surgery was not linked to survival rate. However, morbidity and psychological traumas are detrimental to the quality of life [53]. The absence of minimally invasive therapies and predictive characteristics of penile cancer is an overwhelming burden for medical practice and scientists, who are challenged to search for molecular targets of the disease [54].

Epigenetic mechanisms, like DNA methylation, present great plasticity as the epigenome can be reprogrammed through environmental factors [68, 69]. Aberrant epigenetic reprogramming changes cell landscape given the malignant phenotype, as increased proliferation, resistance to apoptosis, invasion, and metastasis, triggering tumorigenesis [32]. Several studies have been dedicated to the characterization of epimutations in penile SCC [19, 70]. In this study, an increased level of the global 5mC mark was observed regardless of HPV infection. Genomic instability and mutations are important molecular alterations that trigger carcinogenesis. Increased global 5mC (hypermethylation) contributes to genomic stability in tumor progression and, therefore, may be related to tumor invasion and chemotherapy resistance [58, 73]. Unlike our findings, reduced global DNA methylation was reported in other types of the tumors such as colorectal [55] and prostate [56]. As a repressive epigenetic marker, it changes gene expression of specific targets and large chromosomal regions, as repetitive DNA elements [58].

The increased 5mC marker was accompanied by a reduced level of the 5hmc mark in the majority of penile SCC, which was associated with a p16^INK4a^ negative diagnosis. Decreased level of 5hmC mark was observed in head and neck cancers positive for HPV, and virus presence might influence the oxidation process from 5mC to 5hmC in genes of cell junction pathways [65]. 5hmC is an epigenetic marker for active DNA demethylation [57], which depends on TET proteins that mediate this process [71]. Aberrant active DNA demethylation was also observed in non-small cell lung cancer [72]. Furthermore, we observed a complete absence of 5hmC staining in several SCC samples, similar to previous reports about global loss of the 5hmC mark in large subsets of oral squamous cell carcinoma [59] and cervical cancer [60].

Given that 5mC is a protective marker against DNA damage and the hydroxymethylation of 5mC is an active demethylation process, the balance between 5mC and 5hmC marks is directly linked to genomic stability, contributing to cancer development and progression [34]. We observed that a higher 5mC may be a risk factor for death in penile SCC, irrespective of HPV infection, and an increase in sample size may confirm these findings. The 5mC/5hmC mark is an important prognostic and predictive parameter, and we have recently demonstrated the 5mC/5hmC imbalance related to hypercellular bone marrow, dyserythropoiesis and cases of high-risk myelodysplastic syndromes [61]. Moreover, the clinical relevance of 5mC and 5hmC levels was reported as a critical marker for prognosis in colorectal cancer, as increased 5mC was associated with lymph node metastasis [55]. Global epigenetic marks should be deciphered as they indicate characteristics of advanced tumor staging [60] and tumor subtypes biomarker [62], and targeted therapy using DNMT inhibitors [63, 64].

Despite the important findings regarding the HPV incidence and the association with the 5mC/5hmC mark with penile SCC prognosis, other studies should address the epidemiological characteristics of penile cancer and epigenetic reprogramming through lifestyle changes. Our study was limited by the lack of information regarding the participant's background, such as sexual behavior, sociodemographic profile, HPV vaccination, and the identification of other related histological subtypes and tumor topography. Even though we evaluated hrHPV and lrHPV, there is a diversity of viral genotypes that have not been identified individually and should give better knowledge regarding the infection and disease prognosis, as well as the time of exposure to infection that was not tracked. Although partial and total penectomy represents better survival compared to even more invasive ones, these techniques have drastic implications for the individual's personal life, which calls for less invasive therapies. The epidemiologic data, for staging and surgical excision, suggest that medical care is just sought late raising costs in specialized treatment. The elevated incidence of HPV highlights the importance of policies to encourage vaccination to control viral infection in the male population.

## Conclusions

We reported an incidence of 53.2% of hrHPV infection in men with penile SCC in the State of Ceará, Northeast Brazil, a region with the lowest per capita income in the country. Despite the increased incidence, HPV infection was not associated with poor prognosis, such as systemic or lymph node metastasis, locoregional recurrence, but it seems to increase the survival rate. The current research also indicates, for the first time, that increased global DNA 5mC and reduced 5hmC marks are characteristics of penile SCC. Despite no statistical difference, increased 5mC may contribute to poor prognosis as the HR was 1.06 in SCC. Although viral infection contributes to the loss of aberrant DNA reprogramming, the 5mC/5hmC ratio was not related to HPV infection, and 5hmC levels were increased in p16^INK4a^ negative samples. This information should be further explored as this data may predict potential clinical relevance for penile cancer prognosis and may suggest new treatment strategies, as hypomethylating agents for epigenetic targeted therapies (epidrugs). The poor prognosis of penile cancer and its relationship with environmentally induced changes reinforces the benefits of primary health care and the elevated incidence of hrHPV highlights the importance of vaccination and continued educational strategies for both prevention and treatment of malignant lesions.

## Methods

### Participants and study design

This is a retrospective study that included 224 participants with penile SCC who underwent partial or total penectomy, and advantage stage with enlarged prostatectomy and emasculation, without any prior history of chemotherapy or radiotherapy. Penile SCC samples were obtained at Hospital Haroldo Juaçaba, Ceará, Northeast Brazil, and detailed clinicopathological and follow-up data were assessed from 2000 to 2018. The study was approved by our Institutional Review Board (process number 2.427.846).

Available epidemiological data for penile SCC were obtained. The anatomopathological evaluation was performed by two pathologists who were blinded to the clinical data, and the regions corresponding to the neoplasm were marked on the histological slides. Pathological staging was performed according to the eighth edition of the American Joint Committee on Cancer (AJCC) (2017) [36]. Local recurrence in the amputation stump, as well as lymph node and systemic metastasis, was evaluated. The mean follow-up was 29.69 (37.3) months from surgery to the last visit or death. As we had different types of block quality and the amount of material had to be fractionated for pathological analysis and p16^INK4a^, hrHPV, 5mC, and 5hmC markings, the number of samples available for analysis varied (Table 1 and Additional file 1: Supplementary Fig. 1).

### Tissue microarray construction

The Tissue MicroArray (TMA) blocks were composed of representative 2.0 mm cores, in duplicate, from the 224 samples from the epidemiological study cases. Blocks were sectioned to 4 μm thick and mounted on glass slides with an organosilane-based adhesive (3-aminopropyltriethoxy-silane; Sigma Chemical Co®, St Louis, MO, USA). TMA sections were then used for staining in HPV detection assays and analyses of 5mC/5hmC epigenetic markers. All the experiments were analyzed by a blinded observer.

### p16^INK4a^ immunoexpression and HPV in situ hybridization

The p16^INK4a^ immunoexpression was carried out using an anti-p16 antibody clone E6H4 (Roche, USA). p16^INK4a^ expression must be ≥ 75% in neoplastic cells to be considered positive in immunohistochemistry (IHC) staining, with continuous and complete cytoplasmic and nuclear staining [37]. Chromogenic in situ hybridization (CISH) was used to distinguish hrHPV and low-risk HPV (lrHPV) forms. For that, Ventana Inform HPV III Family 16 Probe (Ventana Medical Systems, Tucson, AZ) diagnostic kit was used to identify hrHPV (genotypes 16, 18, 31, 33, 35, 39, 45, 51, 52, 56, 58 and 66) and Ventana Inform HPV II Family 6 (Ventana Medical Systems, Tucson, AZ) for lrHPV identification (genotypes 6 and 11). High-grade cervical intraepithelial neoplasia was used as a positive control for hrHPV and condyloma acuminata for lrHPV. Skeletal striated muscle was used as a negative control for both assays. Representative images of the positive and negative markings of the tumor area for each assay used in this work are available in Additional file 1: Supplementary Fig. 2.

### Global DNA methylation and hydroxymethylation assays

The TMA sections were stained with anti-5-methylcytosine (anti-5mC) and anti-5-hydroxymethylcytosine (anti-5hmC) antibodies using streptoavidin-biotin IHC. Briefly, after deparaffinization and rehydration, TMA slides were submitted to antigenic recovery in citrate pH 6.0 0.01 M. The slides were, then, blocked by endogenous peroxidase in a 3% hydrogen peroxide solution for 30 min, to block unspecified staining. Subsequently, slides were incubated with primary antibodies (anti-5mC: Abcam–Ab214727ug50; anti-5hmC: Abcam–Ab214728ug50) in a humid and dark chamber at 4 °C overnight. Secondary incubation took place with the DAKO EnVision™ kit (DAKO®, Carpentaria, CA, USA) for 30 min at room temperature and counterstained with Mayer’s hematoxylin. The analysis of 5mC and 5hmC was performed by evaluating the percentage of positivity of 100 cells in the tumor area of each TMA sample, in duplicate, for each target. Counting was made by two independent observers and the percent positivity rate was used to construct the score (% score). As each sample had 5mC and 5hmC scores, the 5mC/5hmC ratio was calculated for all participants. We used brain samples as positive control and the absence of the primary antibody during the incubation as the negative control.

### Statistical analyses

Qualitative variables were summarized considering absolute and relative frequencies. The Fisher’s exact test was applied to verify which qualitative variables are associated with p16^INK4a^ and hrHPV and the Wilcoxon nonparametric test for independent variables was applied to verify the relation between quantitative variables and p16^INK4a^ and hrHPV. A multivariate regression analysis of survival data was based on the Cox proportional hazards model using PHREG procedure. The characteristics with reduced sample size as staging (pT4) were not included in the model. The Student's t-test was used to compare 5mC and 5hmC marks and HPV infection. All statistical analyses were performed using SAS® 9.3 software (SAS Institute Inc, University of North Carolina, North Carolina), and *P* < 0.05 was considered significant. The Kaplan–Meier curves were predicted by log-rank (Mantel-Cox) test performed in GraphPad Prism version 8.4.2. For survival curves, the sample size evaluated to time to death is presented in Table 1.

## Supplementary Information

**Additional file 1**. Study flowchart, immunohistochemistry and survival rate for penile squamous cell carcinoma.

## Data Availability

Not applicable.
