# Supplementary material for: HPV infection and 5mC/5hmC epigenetic markers in penile squamous cell carcinoma: new insights into prognostics
Source: Clin Epigenetics. 2022 Oct 25;14:133. doi: 10.1186/s13148-022-01360-1 (PMC9597985; doi:10.1186/s13148-022-01360-1)
Supplement: Supplementary file 1 — Additional file 1. Study flowchart, immunohistochemistry and survival rate for penile squamous cell carcinoma. [file 13148_2022_1360_MOESM1_ESM.docx]

**ADITIONAL FILE:** Study flowchart, immunohistochemistry and survival rate for penile squamous cell carcinoma.

**Supplementary Fig 1**. Flowchart of experimental data for HPV and methylation.


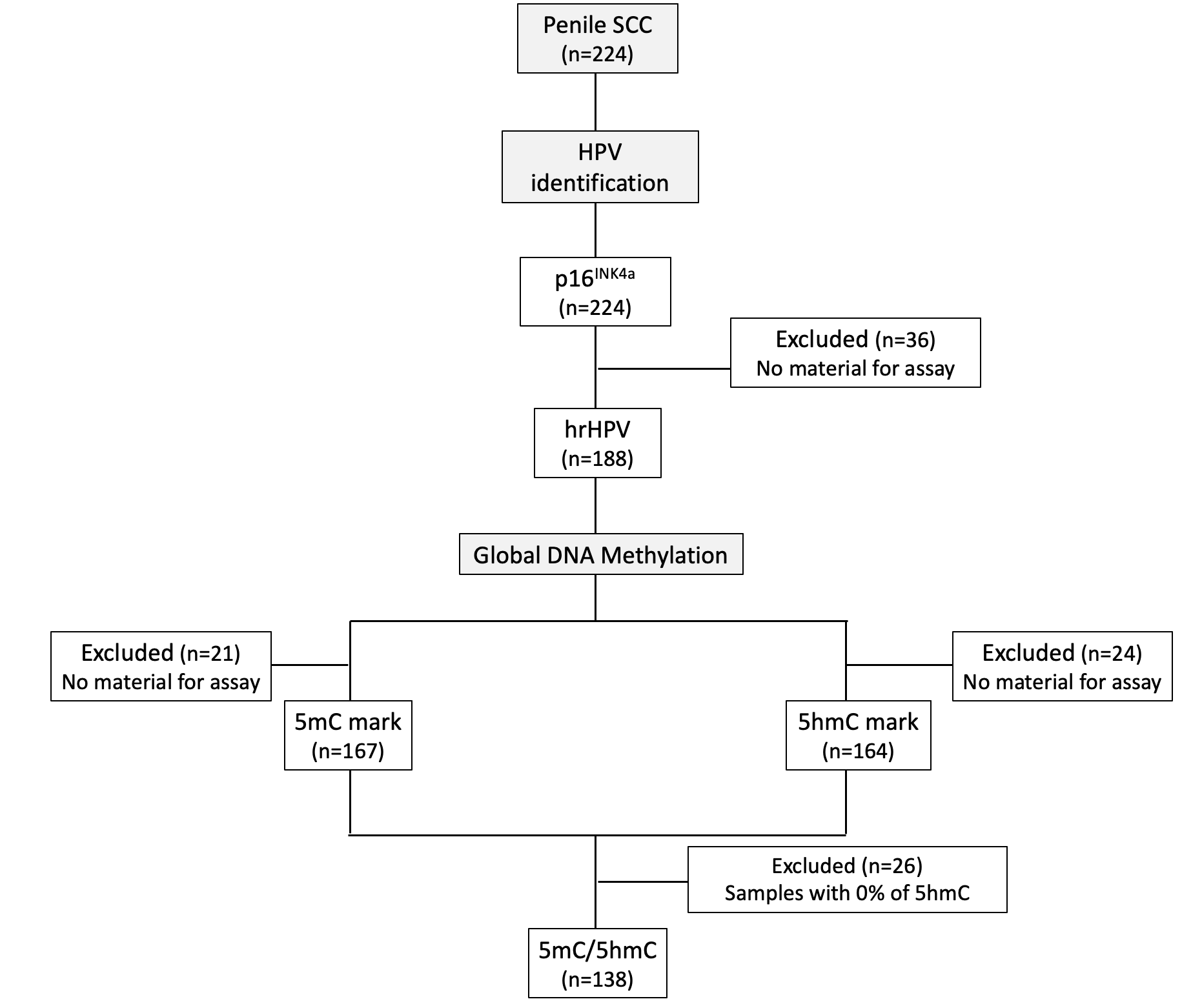


**Supplementary Fig 2**. Representative images for hrHPV hybridization positive (A) and negative (B), p16 immunoexpression positive (C) and negative (D), 5mC mark positive (E) and negative (F), and 5hmC mark positive (G) and negative (H).


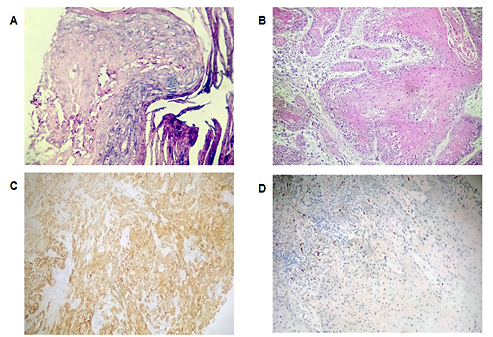


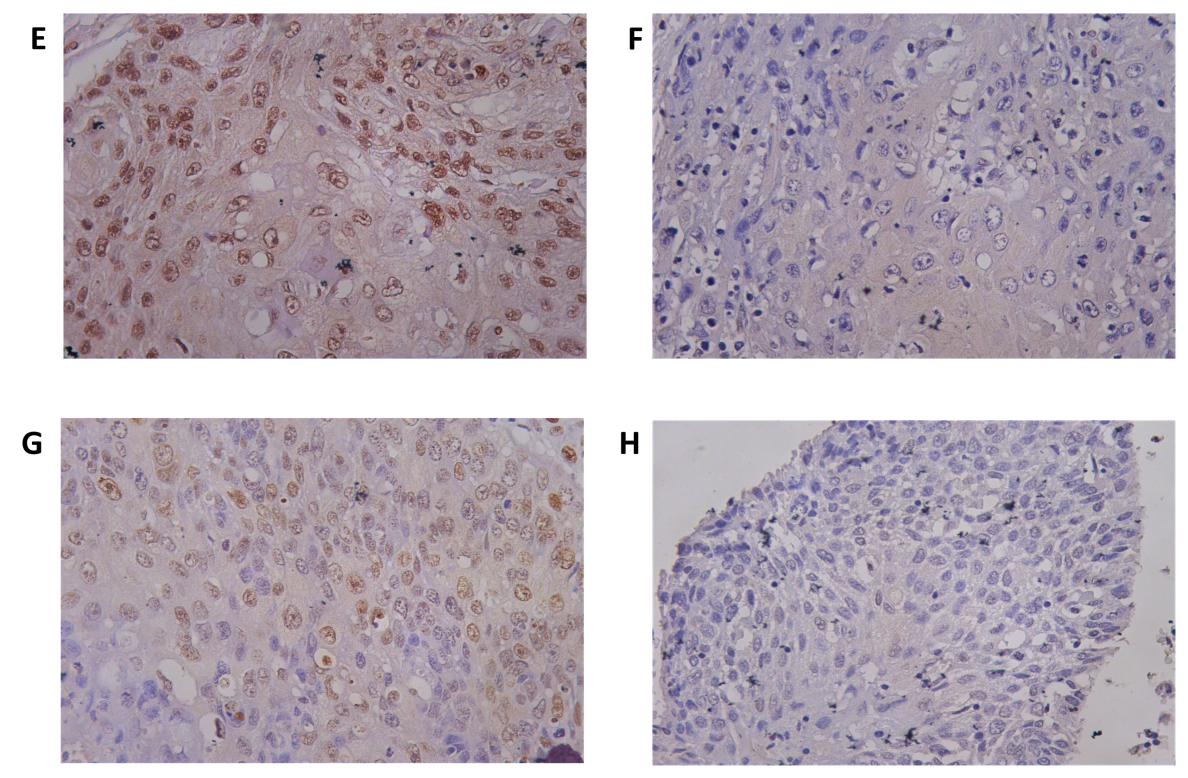


**Supplementary Fig 3**. Survival curve for tumor infiltrate. A, peritumoral lymphocyte infiltrate (PLI); B, peritumoral polymorphonuclear infiltrate (PPI); C, intratumoral lymphocyte infiltrate (ILI), D; intratumoral polymorphonuclear infiltrate (IPI) and staging (S).


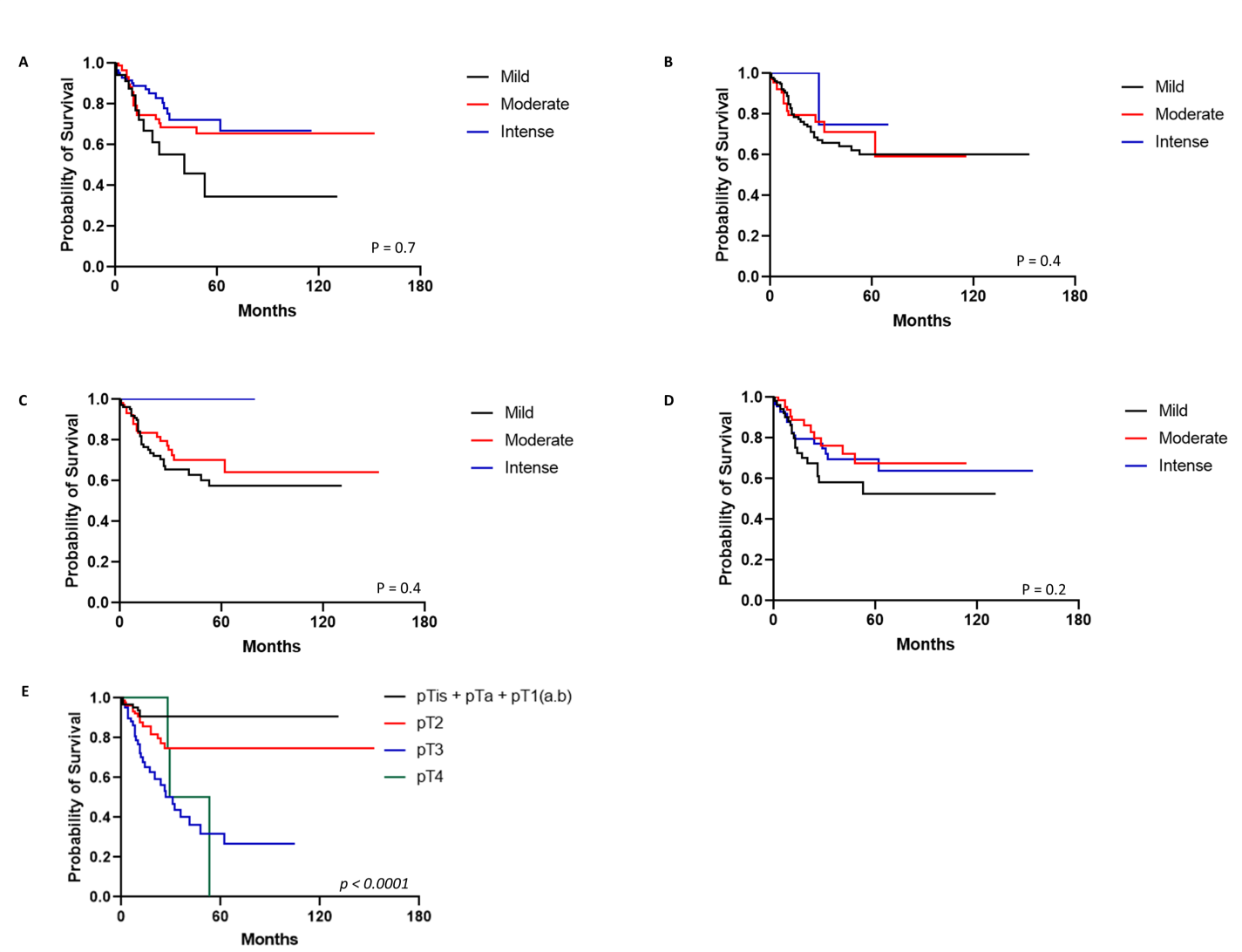


PPI

PLI

IPI

ILI


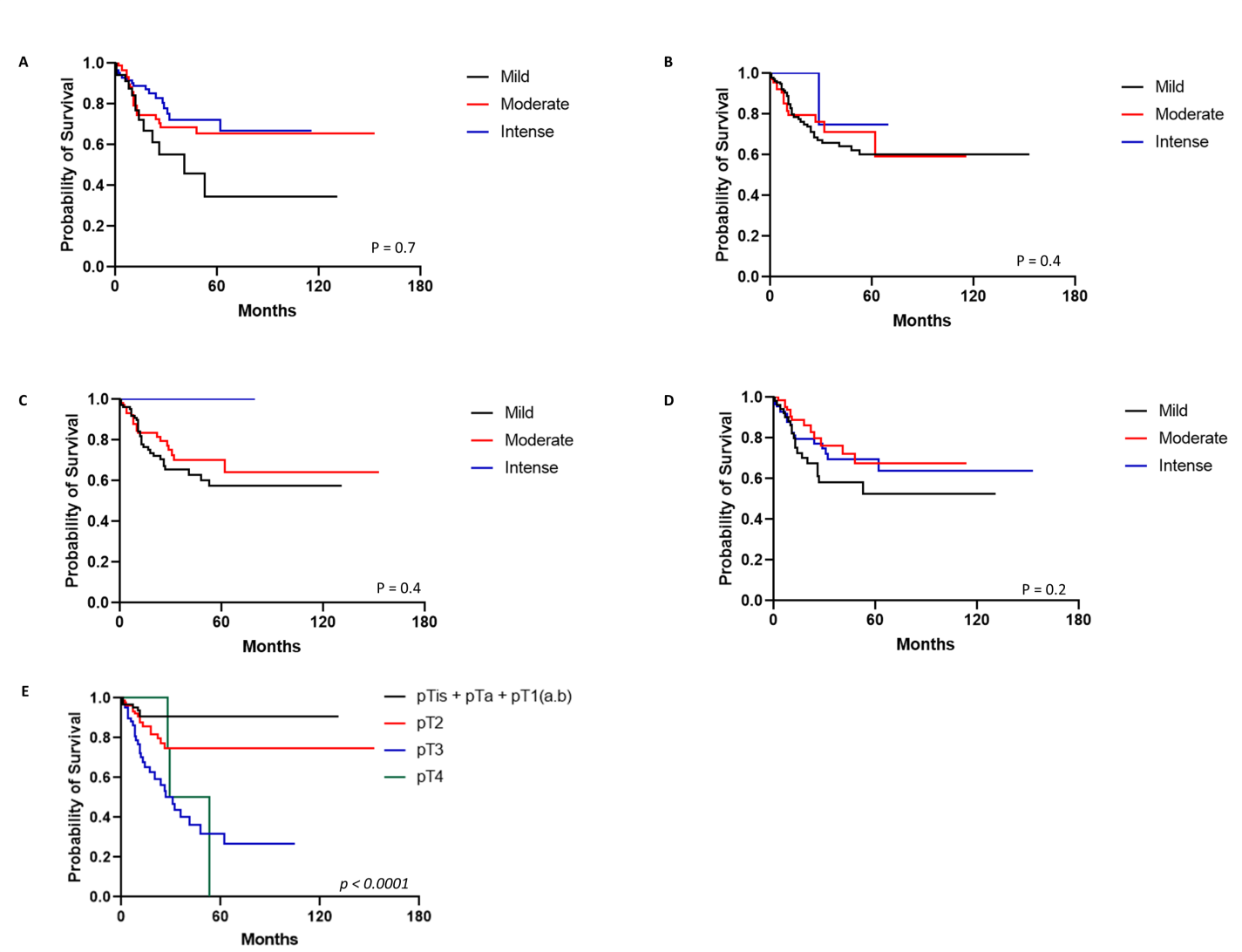


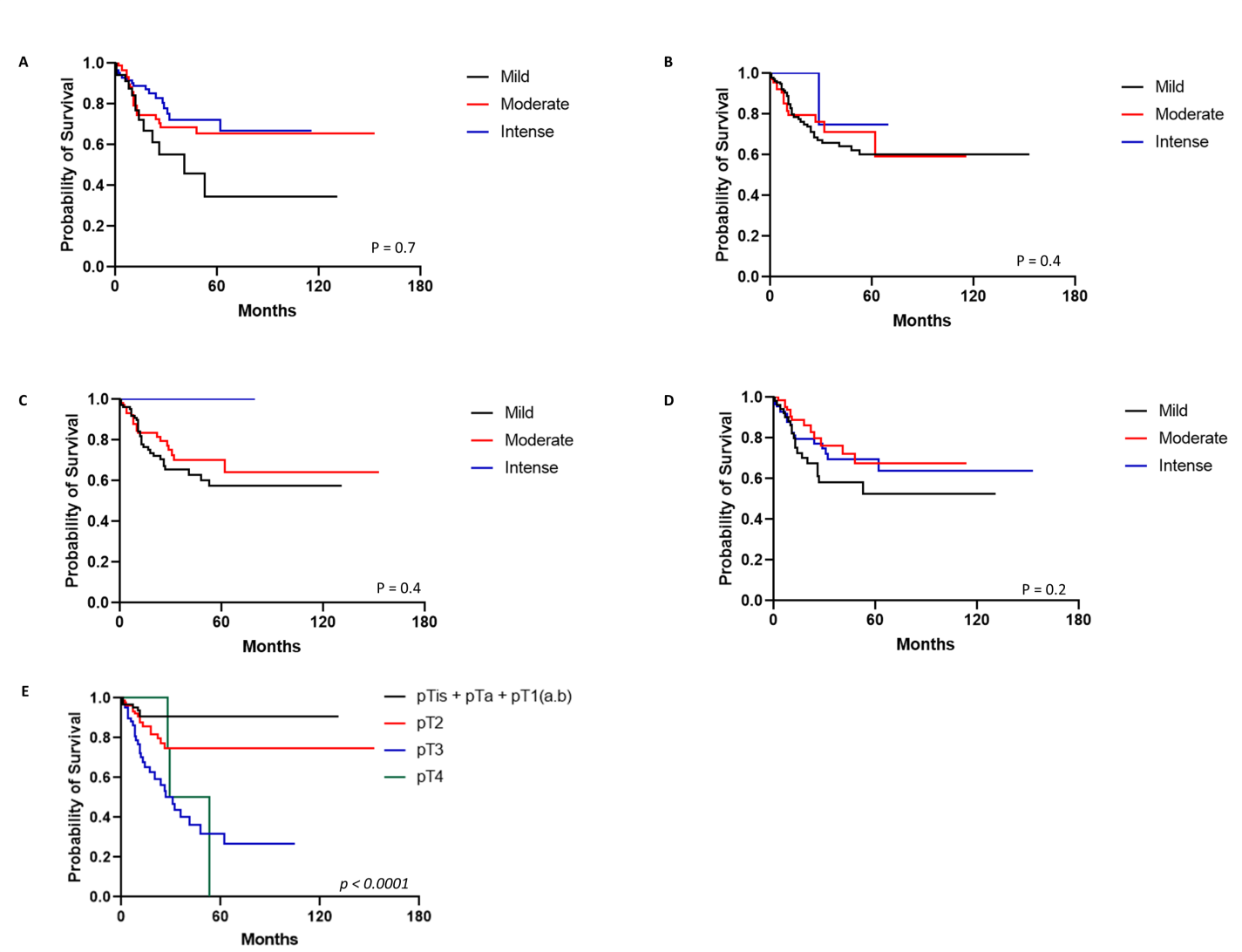


**Supplementary Table 1.** Adjusted Cox Hazard Ratio analysis.

| Variables | | exp(coef) | lower 95 | upper ,95 | Adj-pvalue |
| --- | --- | --- | --- | --- | --- |
| HPV | 0,8219 | | 0,3641 | 1,8554 | 0,636848 |
| P16 | 0,3358 | | 0,1185 | 0,9515 | **0,04002** |
| Lymph node metastasis | 4,6456 | | 1,3747 | 15,6993 | **0,013431** |
| Locoregional recurrence | 5,5179 | | 2,2004 | 13,8369 | **0,000271** |
| Systemic metastasis | 4,3968 | | 1,6822 | 11,4916 | **0,002519** |
| Age | 1,0262 | | 0,9981 | 1,0551 | 0,06746 |
| 5hmC | 0,9487 | | 0,5991 | 1,5022 | 0,822214 |
| 5mC | 1,0588 | | 0,332 | 3,3763 | 0,923097 |
| Chemotherapydei | 1,7263 | | 0,5989 | 4,9764 | 0,312129 |
| Radiotherapy | 1,0377 | | 0,3806 | 2,8296 | 0,94229 |
| Total amputation | 1,1563 | | 0,4704 | 2,8422 | 0,751701 |
| Other surgeries | 3,3079 | | 0,8737 | 12,5243 | 0,07821 |

In bold p<0.05
